# Facilitating text reading in posterior cortical atrophy

**DOI:** 10.1212/WNL.0000000000001782

**Published:** 2015-07-28

**Authors:** Keir X.X. Yong, Kishan Rajdev, Timothy J. Shakespeare, Alexander P. Leff, Sebastian J. Crutch

**Affiliations:** From the Dementia Research Centre, Department of Neurodegeneration, Institute of Neurology (K.X.X.Y., K.R., T.J.S., S.J.C.), Institute of Cognitive Neuroscience (A.P.L.), and Department of Brain Repair and Rehabilitation, Institute of Neurology (A.P.L.), University College London, UK.

## Abstract

**Objective:**

We report (1) the quantitative investigation of text reading in posterior cortical atrophy (PCA), and (2) the effects of 2 novel software-based reading aids that result in dramatic improvements in the reading ability of patients with PCA.

**Methods:**

Reading performance, eye movements, and fixations were assessed in patients with PCA and typical Alzheimer disease and in healthy controls (experiment 1). Two reading aids (single- and double-word) were evaluated based on the notion that reducing the spatial and oculomotor demands of text reading might support reading in PCA (experiment 2).

**Results:**

Mean reading accuracy in patients with PCA was significantly worse (57%) compared with both patients with typical Alzheimer disease (98%) and healthy controls (99%); spatial aspects of passages were the primary determinants of text reading ability in PCA. Both aids led to considerable gains in reading accuracy (PCA mean reading accuracy: single-word reading aid = 96%; individual patient improvement range: 6%–270%) and self-rated measures of reading. Data suggest a greater efficiency of fixations and eye movements under the single-word reading aid in patients with PCA.

**Conclusions:**

These findings demonstrate how neurologic characterization of a neurodegenerative syndrome (PCA) and detailed cognitive analysis of an important everyday skill (reading) can combine to yield aids capable of supporting important everyday functional abilities.

**Classification of evidence:**

This study provides Class III evidence that for patients with PCA, 2 software-based reading aids (single-word and double-word) improve reading accuracy.

Posterior cortical atrophy (PCA) is a progressive syndrome most frequently caused by Alzheimer disease (AD), characterized by posterior atrophy and prominent impairment in visuospatial and visuoperceptual function^1,2^ with relatively spared memory and semantic knowledge.^1,3–5^ A debilitating symptom of PCA is dyslexia (80%–95%^1,3,6^), which is likely peripheral in nature.^7–9^ Clinical reports describe patients with PCA as having difficulty following text along a printed line, getting lost from one line to the next,^10,11^ seeing words in “false order,”^12^ and losing their place on a page^9,13^; however, these have yet to be empirically investigated. In this study, we set out to characterize text reading in PCA (experiment 1) to inform the design of a reading aid for this patient group (experiment 2).

We developed 2 reading aids (single- and double-word) intended to support reading in PCA by minimizing the spatial and oculomotor demands of text reading. Both aids moved words successively into a fixation box; we hypothesized that such presentation would assist in localizing words within sentences and identification of the onset of subsequent lines of text by reducing the susceptibility of text reading to visual disorientation, excessive crowding, and fixation instability.

The current study compared the reading performance of individuals with PCA, those with typical AD (tAD), and healthy controls. Experiment 1 examined the hypothesis that spatial factors are the primary determinants of PCA text reading accuracy. Experiment 2 tested the hypothesis that reducing the spatial and oculomotor demands of text reading would significantly improve reading ability in PCA.

## PARTICIPANTS

Fifteen patients with PCA, 6 patients with tAD, and 6 healthy controls participated in both experiment 1 and 2. Numbers of patients with PCA were limited because of the low prevalence of PCA; small numbers of patients with tAD and healthy control participants reflected expectations of near-ceiling performance while fulfilling counterbalancing requirements for experiment 2 (table e-1 on the *Neurology*® Web site at Neurology.org). Based on clinical and neuroimaging data, the patients with PCA all fulfilled clinical criteria for a diagnosis of PCA^1,3,14^ and research criteria for probable AD.^15^ The patients with tAD fulfilled research criteria for a diagnosis of typical amnestic AD.^15^ Demographic information did not significantly differ among PCA, tAD, and control groups (table 1). Molecular pathology (^18^F amyloid imaging performed as part of another investigation or CSF) was available for 5 of 15 patients with PCA and 4 of 6 patients with tAD. All results were consistent with AD pathology (positive amyloid scan on standard visual rating or CSF β-amyloid [Aβ]_1–42_ ≤450 and/or tau/Aβ ratio >1), with PCA and tAD groups fulfilling criteria for atypical and tAD, respectively.^16^

**Table 1:**
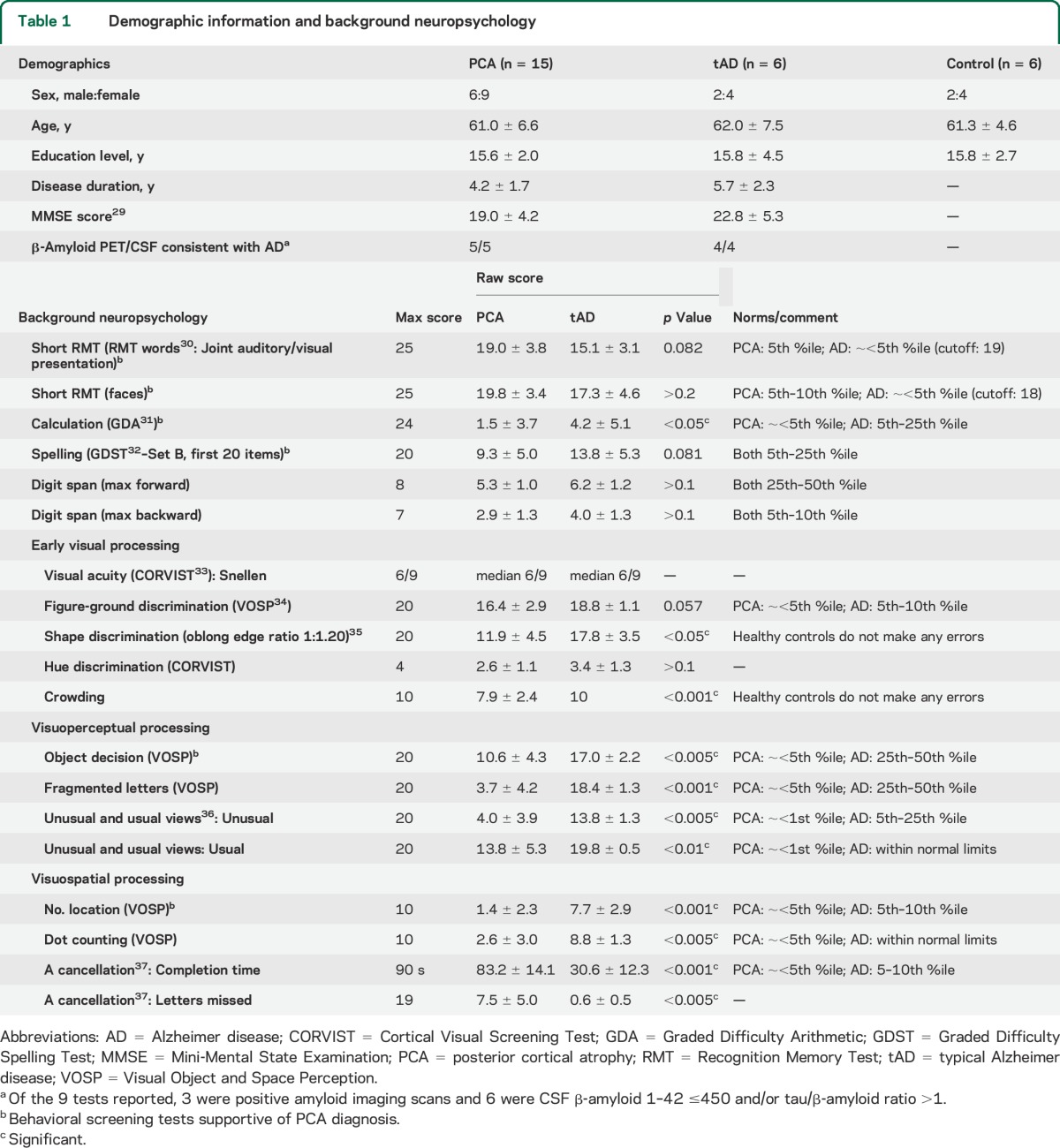
Demographic information and background neuropsychology

### Standard protocol approvals, registrations, and patient consents.

Ethical approval for the study was provided by the National Research Ethics Service London–Queen Square ethics committee and written informed consent was obtained from all participants.

## BACKGROUND NEUROPSYCHOLOGY

A battery of neuropsychological tests was administered to both patients with PCA and patients with tAD. Mean scores on each test and an estimate of their performance relative to normative datasets are shown in table 1. Overall PCA performance was consistent with cognitive characteristics outlined in previous studies,^1,3,16^ with most prominent symptoms being higher-order visual deficits with relatively less impaired memory ability.

## EXPERIMENT 1: EFFECT OF SPATIAL FACTORS ON TEXT READING

### Methods.

#### Stimuli.

Participants read aloud 6 text passages (mean word count: 107.0, SD = 5.2) in a standard presentation block-of-text format (mean number of lines: 14.8, SD = 1.2), with each passage split into 3 paragraphs. Passages were selected from the BBC news archive to reduce priming from current events. Words were in black Arial Unicode MS font size 16, with a visual angle of letter height subtending 0.45° when viewed from a distance of 50 cm, presented on a gray background.

#### Procedure and apparatus.

Participants were given a maximum of 300 seconds to read each passage. For each word, participants who took more than 10 seconds to respond were prompted to move onto the next word. Participants were not discouraged from using their finger to maintain their place when reading. Passages were administered through a repeated-measures design that included presentation conditions from experiment 2 (see below). Latencies were defined as the time taken to read each passage and were manually determined from when passages were first presented. Words read correctly were marked as accurate, regardless of word order. An Eyelink II (SR Research Ltd., Mississauga, Canada) recorded gaze location at 250 Hz.

#### Data analysis.

Between-group differences in reading accuracy, reading speed, and performance on neuropsychological measures were calculated using Wilcoxon rank sum tests. Three separate models analyzed spatial influences on reading accuracy (table 2).

**Table 2:**
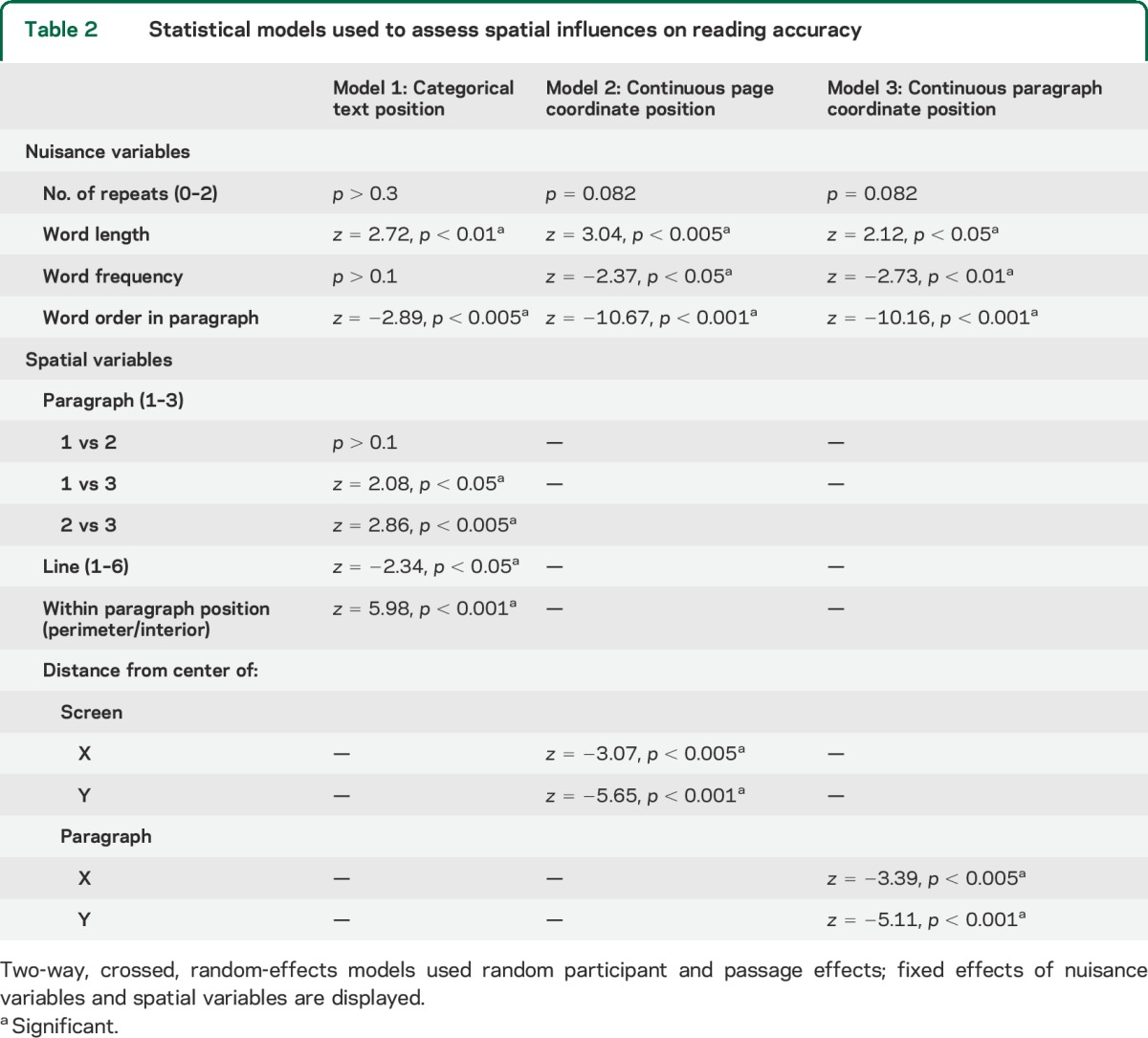
Statistical models used to assess spatial influences on reading accuracy

##### Eye movement data.

Between-group differences in fixation number/duration and saccade number/amplitude were assessed using Wilcoxon rank sum tests.

### Results.

#### Reading performance.

**Figure 1:**
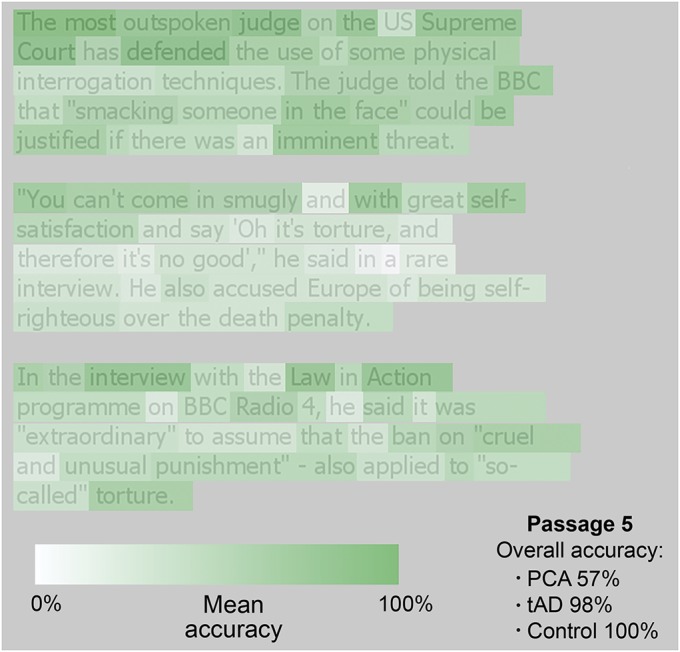
Heatmap of PCA group accuracy data from a sample passage Heatmap of PCA accuracy data from a sample passage (mean group accuracy rates: PCA = 57%, tAD = 98%, controls = 100%). Lighter colors indicate the location of words most frequently missed or misread, and indicate spatial biases toward worse reading in later paragraphs and lines and in the center of dense, crowded passages of text. PCA = posterior cortical atrophy; tAD = typical Alzheimer disease.

**Figure 2:**
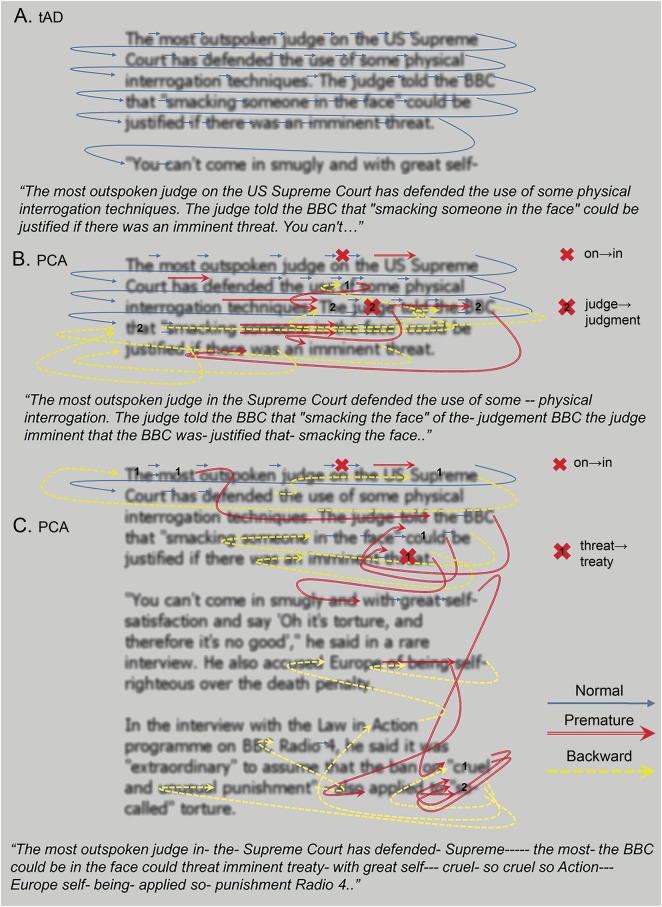
Word order from a sample passage for a patient with tAD and 2 patients with PCA Order of first 40 words read by (A) a representative patient with tAD; (B) a patient with PCA who had an MMSE score = 13/30; and (C) a patient with PCA who had an MMSE score = 22/30. Arrows outline reading order; red arrows indicate omission of subsequent words through reading later sections of text, and yellow arrows indicate reading of earlier sections of text. Transcripts of the participants' corresponding spoken output are beneath each example (italicized text). Each hyphen in the transcript beneath each example indicates a pause of 3 seconds. Numbers refer to where words were repeated. Under our marking scheme, words were marked as correct regardless of word order: in this way, the participant (C) was considered to have read 38 of her first 40 words correctly. Thus, accuracy rates constitute, if anything, an overestimate of reading ability. MMSE = Mini-Mental State Examination; PCA = posterior cortical atrophy; tAD = typical Alzheimer disease.

A heatmap showing the effect of word location on PCA reading accuracy for an example passage is shown in figure 1. Patients with PCA were less accurate overall (57.2%, SD = 21.7) than patients with tAD (97.5%, SD = 2.4; *z* = −3.43, *p* < 0.001) and controls (99.4%, SD = 0.01; *z* = 3.51, *p* < 0.001); there was a trend toward tAD patients reading less accurately than controls (*z* = 1.93, *p* = 0.054). Overall accuracy would have been lower if word order had been considered, particularly in the PCA group, because words were marked correct regardless of when they were read in each passage (figure 2). Mean passage reading time was much slower in the PCA group (174.0 seconds, SD = 73.9) than in the tAD group (52.0 seconds, SD = 18.3; *z* = 3.50, *p* < 0.001) and control group (36.2 seconds, SD = 5.2; *z* = 3.50, *p* < 0.001), with the tAD group also slower than controls (*z* = 2.08, *p* < 0.05).

#### Spatial influences on accuracy.

Patients with PCA were more accurate reading words further from the center of the page or paragraph, words on paragraph perimeters, on lines at the beginning of paragraphs, and in earlier paragraphs (table 2).

There were no significant effects of any of the spatial variables on reading accuracy of patients with tAD. The control group did not make enough errors to allow for an accuracy analysis.

In total, 4 patients adopted a strategy to maintain their place when reading by using their finger: 3 patients with PCA (mean accuracy: 47.1%, SD = 17.1; mean latency: 164.2 seconds, SD = 100.7) and one patient with tAD (99.5%; 87 seconds). The patients with PCA who used their finger did not significantly differ in reading speed (*p* > 0.6) or accuracy (*p* > 0.3) from the remainder of the PCA group (n = 12; 59.7%, SD = 22.7; 176.5 seconds, SD = 71.3); a modified *t* test^17^ found that the patient with tAD who used their finger was slower (*t* = 5.42; *p* < 0.01) but not less accurate (*p* > 0.4) than the remainder of the tAD group (n = 5; 97.1%, SD = 2.51; 45.0 seconds, SD = 7.1).

#### Eye movement data.

Saccade and fixation data are shown in table e-2. Patients with PCA made significantly more saccades, fixations, and had longer fixation durations than controls and patients with tAD. Patients with PCA made significantly more horizontal saccades than controls and left saccades than patients with tAD, but saccade amplitude did not differ significantly. There were no significant differences in any eye movement measures between the tAD and control group.

## EXPERIMENT 2: FACILITATING READING IN PCA

### Methods.

#### Stimuli.

To contrast with the baseline condition of standard text reading (as described in experiment 1), 2 reading aids were designed to provide the optimal conditions for reading in PCA by minimizing the spatial, perceptual, and oculomotor demands of reading: (1) single-word: passages presented one word at a time in a central fixation box; and (2) double-word: identical to the single-word condition except that each target word was accompanied by the subsequent word, displayed parafoveally to the right of the fixation box (mean center-to-center distance between target/parafoveal word: 5.7°).

**Figure 3:**
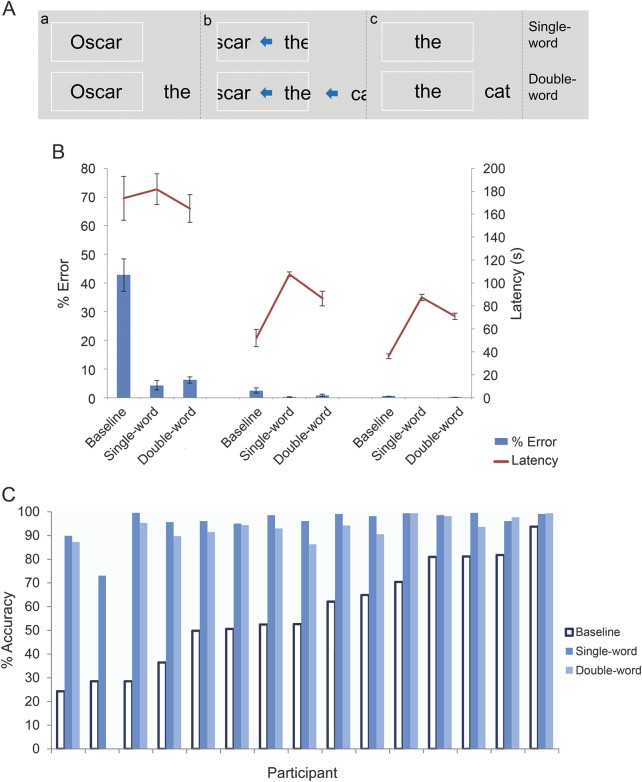
Single- and double-word presentations, reading performance in patient/control groups, and individual PCA patients' reading accuracy (A) Single-word and double-word presentations: words appear in the fixation box (a); following participants' responses, successive words move rapidly (b) into the fixation box (c). (B) Summary of reading accuracy (percentage error) and latencies (seconds) for the PCA, tAD, and control groups. Error bars show standard error for each group mean. (C) Reading accuracy of participants with PCA (percentage correct) for baseline (standard presentation) and under both reading aids, ordered by baseline severity. PCA = posterior cortical atrophy; tAD = typical Alzheimer disease.

In both conditions, words were successively moved at a velocity of 22.8°/s from a location 5.7° to the right of the fixation box into the center of the fixation box intended to limit visual disorientation. Restricting text presentation to individual words was intended to evade crowding effects from adjacent words.^18,19^ This motion was intended to act as a cue to assist disorientated readers, following reports of patients with PCA being able to better localize moving stimuli.^20,21^ The double-word condition in addition presented the subsequent word in each passage to the right of the fixation box in order to assess whether participants' reading would benefit from the presence of words to the right of fixation.^22,23^ Words remained stationary within the fixation box until read; subsequently, consecutive words were moved into the box by the experimenter (figure 3A). The fixation box (height: 2.1°; width: 4.3°) was white (opposite polarity to text) to limit any crowding effects of the box on word identification.

#### Procedure.

Participants read aloud the 6 passages described in experiment 1 under 3 conditions: baseline (standard text presentation: experiment 1), single-word, and double-word. Each passage was read in every condition by each participant (in the same order) in a repeated-measures design to control for lexical and syntactic differences between passages. All 3 presentation conditions were arranged in 1 of 6 variants of a Latin Square design within 3 blocks each composed of 6 passages, with presentation condition always differing between each passage; this was done to avoid confounding differences between presentation conditions and practice effects. The order of these blocks varied across participants to control for passage effects (table e-1). Because of time constraints, one patient with PCA read passages under baseline and single-word, but not double-word, conditions. Controls completed a third of the trials, with no passage repeats and varying presentation order. After each passage, patients with PCA rated their reading ease, comprehension, and pleasantness on a 4-point auditory-verbal scale (very, quite, not really, not at all). For the first 3 passages, PCA and tAD groups were also asked a global comprehension question (“Can you tell me the gist of that article?”) to ensure that participants were extracting semantic information from each passage. Eye movements were recorded as in experiment 1.

#### Data analysis.

Regression models of accuracy and eye movement analyses were identical to those in experiment 1 except spatial variables were replaced with presentation condition. Regression models were used to test for an interaction between patient group and presentation condition. Global comprehension question responses were assigned by blinded raters (n = 14) to the appropriate corresponding paragraph. Differences in global comprehension and self-rated ease, comprehension, and pleasantness measures were compared within- and between-group using Wilcoxon signed rank and rank sum tests.

#### Classification of evidence.

This study provides Class III evidence on the primary research question of whether 2 reading aids, single- and double-word, improve reading accuracy in patients with PCA.

### Results.

#### Efficacy of reading aids.

Percentage error rates and reading latency data for each presentation condition are shown in figure 3B. There was an interaction between patient group and condition; patients with PCA performed significantly more accurately than patients with tAD in the single-word (*z* = 3.62, *p* < 0.001) and double-word (*z* = 5.81, *p* < 0.001) relative to the baseline condition. There was no significant interaction between group (PCA vs controls) and condition because of near-ceiling performance by controls (overall error rate: 0.3%).

##### PCA.

Relative to baseline, PCA reading accuracy was significantly higher in both single-word (67% improvement; *z* = 38.17, *p* < 0.001) and double-word (64% improvement; *z* = 34.82, *p* < 0.001) conditions. These improvements were evident in every individual patient with PCA (figure 3C). The PCA group performed more accurately in the single- than double-word condition (*z* = −5.61, *p* < 0.001). Accuracy was lower for words read later in each passage (*z* = −11.35, *p* < 0.001) and words of higher frequency (*z* = −2.3, *p* < 0.05). There were no effects of repeats (*p* > 0.6) or word length (*p* > 0.2). There was no significant difference in reading latency between the baseline condition and single-word (*p* > 0.3) or double-word (*p* > 0.7) conditions.

##### tAD.

Relative to baseline, patients with tAD showed modest increments in accuracy with the single-word (2.3%; *z* = 4.60, *p* < 0.001) and double-word (1.7%; *z* = 3.24, *p* < 0.005) conditions. Only 2 of 6 and 1 of 6 patients with tAD improved significantly with the single-word and double-word condition, respectively; the greatest increase in accuracy for a patient with tAD was from 93% to 99%. The tAD group performed more accurately in the single-word than double-word condition (*z* = 2.00, *p* < 0.05). There were no effects of repeated passages (*p* > 0.1), word length (*p* > 0.3), frequency (*p* > 0.3), or word order (*p* > 0.4). Reading speed was fastest in the baseline relative to both the single- and double-word conditions (both *z* = 2.20, *p* < 0.05), and faster in the double-word than single-word condition (*z* = 1.99, *p* < 0.05).

##### Controls.

Reading accuracy was near-ceiling in all 3 conditions. As with patients who had tAD, reading speed was faster in the baseline than single-word or double-word condition (both *z* = 2.20, *p* < 0.05), and faster in the double-word than single-word condition (*z* = 2.21, *p* < 0.05).

#### Self-reported measures.

See figure e-1 for self-reported measures of reading experience in patients with PCA, who rated single-word and double-word reading as significantly easier (*z* = 3.41, *p* < 0.001; *z* = 3.30, *p* < 0.005), more pleasant (*z* = 3.24, *p* < 0.005; *z* = 2.58, *p* < 0.05), and more readily understood (*z* = 3.38, *p* < 0.001; *z* = 3.14, *p* < 0.005) than in the baseline condition. Patients with PCA also rated single-word reading as significantly easier (*z* = 2.18, *p* < 0.05) and more pleasant (*z* = 2.15, *p* < 0.05) but not better understood (*p* > 0.2) than double-word reading.

#### Global comprehension.

Neither patients with PCA nor patients with tAD showed within-group differences in global comprehension across baseline, single-word, and double-word conditions (PCA: 90.9%, 96.2%, and 88.3%; tAD: 86.9%, 72.6%, and 82.1%, respectively). However, patients with PCA showed a trend toward better global comprehension than patients with tAD in the single-word condition (*z* = 1.82, *p* = 0.068) but not baseline (*p* > 0.2) or double-word (*p* > 0.5) condition.

#### Eye movement data.

Saccade and fixation data for each condition are shown in table e-2. As with the baseline condition (experiment 1), patients with PCA made significantly more saccades and fixations than patients with tAD or controls in both single-word and double-word conditions. However, the single-word condition was the only condition in which PCA fixation durations did not differ significantly from patients with tAD or controls, representing an increase in fixation duration relative to the baseline condition (349 ± 42 milliseconds vs 467 ± 71 milliseconds; *z* = 2.20, *p* < 0.05). Single-word reading also led to decreased saccade amplitude relative to the baseline condition (*z* = −1.99, *p* < 0.05).

## DISCUSSION

The current investigation was aimed first to characterize and second to ameliorate text reading deficits in PCA. Experiment 1 demonstrated the debilitating effects of spatial location on text reading in PCA. Each of the 7 spatial dimensions assessed were found to predict passage reading accuracy in PCA, but not in tAD or healthy control participants: patients with PCA were less accurate at reading words positioned within rather than on the edge of paragraphs, toward the end of paragraphs, and in the second or third rather than the first paragraph of each passage. Patients with PCA were also less accurate at reading words positioned toward the center of each paragraph or the overall passage. Results illustrate the deficits undermining word localization in PCA and particular difficulties locating words within a body of text; these deficits are likely a consequence of visuospatial impairment, a restriction in the effective field of vision,^6,20,24,25^ and crowding.^26^

Eyetracking data emphasize the disordered and inefficient quality of text reading in PCA. Despite having much poorer reading accuracy than patients with tAD or control participants, patients with PCA made more fixations and saccades and showed increases in fixation duration; increases in eye movements and fixations likely relate to the slower reading speed of patients with PCA. Qualitatively, some participants' complaints of text moving in the baseline task are consistent with previous reports of perceived motion of static stimuli in patients with PCA, a phenomenon that has been attributed to fixation instability.^9^ An absence of the parafoveal preview benefit,^23^ possibly due to diminished perception of peripheral vision,^6,9,20^ might also account for the increase in fixation duration in the PCA group.

Experiment 2 evaluated the efficacy of 2 reading aids, single- and double-word presentation. Both reading aids resulted in dramatic improvements in the reading accuracy of patients with PCA: single-word presentation in particular resulted in a mean increase of 67.3% in reading accuracy. These gains were accompanied by improvements in self-reported ease of reading, reading comprehension, and pleasantness. Evidently, the serial presentation used in both interventions precludes the otherwise frequent difficulties experienced by patients with PCA in repeating or skipping lines of text and may have reduced the susceptibility of the patients' reading ability to deficits in early or spatial vision. The decrease in saccadic amplitude and increase in fixation duration under the single-word condition suggests a reduction in oculomotor demands and/or improvements in fixation stability, but these demands were not completely abolished as patients with PCA continued to show elevated numbers of saccades and fixations.

There are several potential limitations regarding the current data. Despite pronounced improvements in reading accuracy of patients with PCA under single- or double-word presentation, there was no significant improvement on an independently rated measure of global text comprehension. Strong performance on this measure under baseline (90.9%), single-word (96.2%), and double-word (88.3%) conditions suggests that this lack of a significant improvement might be attributed to ceiling effects. We propose that the efficacy of any home-based reading intervention modeled on the current aids rests not solely on objective measures of reading, but is particularly contingent on its perceived utility and user-friendliness. Consequently, we believe this absence of a significant improvement is offset by other reliable improvements in self-rated measures of reading ability and comprehension. A related potential concern, that global aspects of passage processing are lost under reading aid conditions, is not substantiated when considering both objective and subjective comprehension measures. A second potential issue is that near-ceiling accuracy in the tAD group prevented the identification of factors that reduced reading ability in patients with tAD relative to control participants. Third, despite improvements in reading accuracy, neither reading aid resulted in increases in reading speed. Potential reading applications based on the current aids might allow more efficient rates of presentation maintained by users rather than the experimenter. Because apraxia is a common symptom of PCA,^1,14^ such applications would require the determination of presentation rate using auditory in addition to motor information.

The current investigation demonstrates how the neurologic characterization of a neurodegenerative syndrome (PCA) and a detailed cognitive analysis of an important everyday task (reading) can be combined to yield an aid capable of supporting continued functional ability in patients living with that condition. Both reading aids resulted in robust increases in reading ability and subjective increases in reading ease, comprehension, and pleasantness. Eyetracking data suggested that, in particular, the single-word reading aid promotes a greater efficiency of eye movements. Although some eye movement–based reading therapies result in improvements in reading ability that persist across time and generalize to material encountered outside of the training program,^27,28^ we regard the current interventions as reading aids rather than reading rehabilitation: an aid does not influence the underlying impairment (without glasses, visual acuity decreases to baseline), while rehabilitation does. We plan to develop these aids into assistive technology to support reading ability in patients with PCA and other individuals with dementia-related visual dysfunction.

## Supplementary Material

Data Supplement

## GLOSSARY

Aβ: β-amyloid
AD: Alzheimer disease
PCA: posterior cortical atrophy
tAD: typical Alzheimer disease
