# Supplementary material for: Facilitating text reading in posterior cortical atrophy
Source: Neurology. 2015 Jul 28;85(4):339–48. doi: 10.1212/WNL.0000000000001782 (PMC4520813; doi:10.1212/WNL.0000000000001782)
Supplement: Data Supplement [file supp_85_4_339__index.html]

Data Supplement 

# Facilitating text reading in posterior cortical atrophy

## Data Supplement

Two tables and one figure; two Microsoft Word files and one PDF file.

**Neurology® data supplements are not copyedited before publication. Published editorials and translations have been copyedited.  
 © 2015 American Academy of Neurology.  
  
 Files in this Data Supplement:**

- Figure e-1 - PDF file
- Table e-1 - Microsoft Word file
- Table e-2 - Microsoft Word file
