# Supplementary material for: Facilitating text reading in posterior cortical atrophy
Source: Neurology. 2015 Jul 28;85(4):339–48. doi: 10.1212/WNL.0000000000001782 (PMC4520813; doi:10.1212/WNL.0000000000001782)

Figure e-1. Proportion of passages under the different presentation conditions that were considered easy, pleasant or well understood by PCA patients.

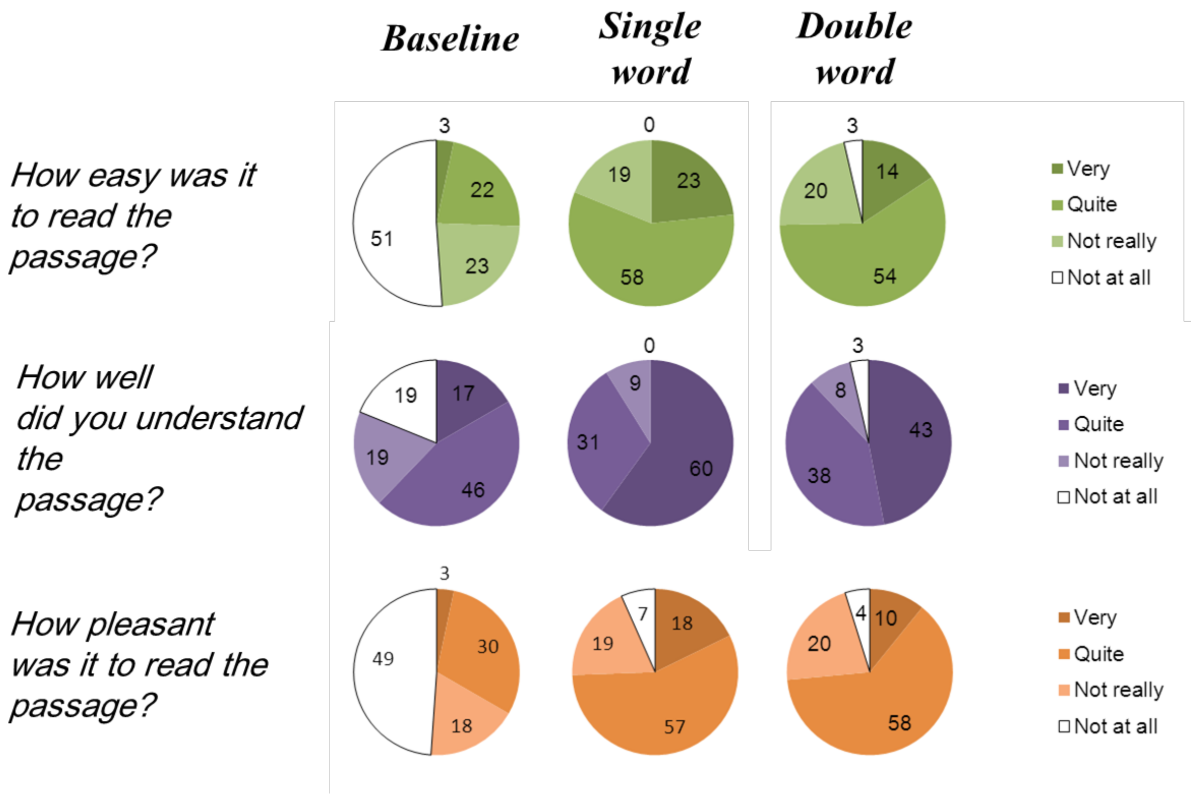

Supplement: Data Supplement [file supp_WNL.0000000000001782_Figure_e-1.pdf]
