# Supplementary material for: Facilitating text reading in posterior cortical atrophy
Source: Neurology. 2015 Jul 28;85(4):339–48. doi: 10.1212/WNL.0000000000001782 (PMC4520813; doi:10.1212/WNL.0000000000001782)
Supplement: Data Supplement [file supp_WNL.0000000000001782_Table_e-1.docx]

| *Passage* | **Participant 1** | **Participant 2** | **Participant 3** | **Participant 4** | **Participant 5** | **Participant 6** |
| --- | --- | --- | --- | --- | --- | --- |
| *1* | B | B | D | D | S | S |
| *2* | S | D | B | S | D | B |
| *3* | D | S | S | B | B | D |
| *4* | B | B | D | D | S | S |
| *5* | S | D | B | S | D | B |
| *6* | D | S | S | B | B | D |
| *1* | S | D | B | S | D | B |
| *2* | D | S | S | B | B | D |
| *3* | B | B | D | D | S | S |
| *4* | S | D | B | S | D | B |
| *5* | D | S | S | B | B | D |
| *6* | B | B | D | D | S | S |
| *1* | D | S | S | B | B | D |
| *2* | B | B | D | D | S | S |
| *3* | S | D | B | S | D | B |
| *4* | D | S | S | B | B | D |
| *5* | B | B | D | D | S | S |
| *6* | S | D | B | S | D | B |

Supplementary Table e-1. Counterbalancing of presentation condition order within- and between-participants.

B=Baseline

S= Single-word

D=Double-word
