# Supplementary material for: Facilitating text reading in posterior cortical atrophy
Source: Neurology. 2015 Jul 28;85(4):339–48. doi: 10.1212/WNL.0000000000001782 (PMC4520813; doi:10.1212/WNL.0000000000001782)
Supplement: Data Supplement [file supp_WNL.0000000000001782_Table_e-2.docx]

Table e-2. Eye movement data for PCA, tAD and control groups in the different presentation conditions. Asterisks denote group differences (vs controls: * = p<.05; **= p<.005; vs tAD:^=p<.05 ).

|  | Saccades | | | | Fixations | |
| --- | --- | --- | --- | --- | --- | --- |
|  | *Overall N* | *Left* | *Right* | *Amplitude* | *Overall N* | *Duration(ms)* |
| Standard paragraph presentation (Baseline) | | | | | | |
| *PCA* | **384 ± 13**^** | **118 ± 29**^** | **166 ± 19**** | 2.0 ± 0.5 | **405 ± 125**^** | **349 ± 42**^** |
| *tAD* | 180 ± 72 | 47 ± 19 | 98 ± 33 | 2.1 ± 0.5 | 190 ± 70 | 254 ± 34 |
| *Control* | 134 ± 22 | 34 ± 11 | 80 ± 9 | 2.3± 0.4 | 141 ± 22 | 239 ± 33 |
| Single-word presentation | | | | | | |
| *PCA* | **336 ± 52**^** | **111 ± 21**^** | **131 ± 53*** | **1.2 ± 0.5^** | **355 ± 49**^** | 467 ± 71 |
| *tAD* | 197 ± 56 | 50 ± 19 | 69 ± 33 | 0.8 ± 0.2 | 214 ± 55 | 544 ± 168 |
| *Control* | 152 ± 50 | 36 ± 23 | 69 ± 28 | 0.8 ± 0.2 | 176 ± 52 | 533 ± 152 |
| Double-word presentation | | | | | | |
| *PCA* | **383 ± 63**^** | **141 ± 42*^** | **168.0 ± 33.2**** | 2.1 ± 0.8 | **402 ± 68**^** | **393 ± 65^** |
| *tAD* | 255 ± 68 | 91 ± 31 | 132 ± 32 | 2.4 ± 1.0 | 264 ± 65 | 305 ± 52 |
| *Control* | 186 ± 46 | 65 ± 25 | 102 ± 20 | 2.5 ± 0.6 | 199 ± 47 | 360 ± 81 |
